# Influência da cobertura de atenção básica e das visitas domiciliares na detecção de casos de tuberculose na cidade do Rio de Janeiro, Brasil, 2014-2022

**DOI:** 10.1590/0102-311XPT056324

**Published:** 2025-02-28

**Authors:** Fernanda de Alencar Lopes, Fernanda Carvalho de Queiroz Mello, Camila Silveira Barbosa, Gledson Felippe, Raquel de Vasconcellos Carvalhaes de Oliveira, Marcel de Souza Borges Quintana, Yara Hahr Marques Hökerberg

**Affiliations:** 1 Escola Nacional de Saúde Pública Sergio Arouca, Fundação Oswaldo Cruz, Rio de Janeiro, Brasil.; 2 Secretaria Municipal de Saúde do Rio de Janeiro, Rio de Janeiro, Brasil.; 3 Instituto de Doenças do Tórax, Universidade Federal do Rio de Janeiro, Rio de Janeiro, Brasil.; 4 Instituto Nacional de Infectologia Evandro Chagas, Fundação Oswaldo Cruz, Rio de Janeiro, Brasil.; 5 Faculdade de Medicina, Universidade Estácio de Sá, Rio de Janeiro, Brasil.

**Keywords:** Tuberculose, Estudos de Séries Temporais, Vigilância Epidemiológica, Aplicações da Epidemiologia, Estratégia Saúde da Família, Tuberculosis, Time Series Studies, Epidemiologic Surveillance, Uses of Epidemiology, Family Health Strategy, Tuberculosis, Estudios de Series Temporales, Vigilancia Epidemiológica, Usos de la Epidemiología, Estrategia de Salud Familiar

## Abstract

Apesar dos esforços para a redução da incidência, o controle da tuberculose (TB) ainda representa um desafio para a cidade do Rio de Janeiro, Brasil. O objetivo deste artigo é analisar a tendência temporal e o efeito das ações de vigilância de TB e do acesso aos serviços de saúde na detecção de casos de TB na cidade do Rio de Janeiro (2014-2022). Realizou-se um estudo ecológico que avaliou todos os casos de TB notificados mensalmente em residentes da cidade do Rio de Janeiro. Foi elaborado um modelo de regressão segmentada de *joinpoint* para identificar pontos de mudança na tendência de notificações e calcular a variação percentual mensal. Modelos aditivos generalizados foram utilizados para avaliar o efeito da implantação do teste rápido molecular, das ações de vigilância de TB e da cobertura de atenção primária na detecção de casos de TB. De janeiro de 2014 a dezembro de 2022, houve uma mediana de 677 casos de TB por mês, com uma variação mensal ascendente, de 0,49% (IC95%: 0,19; 0,79) de janeiro de 2014 a agosto de 2017 e de 0,72% (IC95%: 0,16; 1,29) de dezembro de 2020 a dezembro de 2022. Nos modelos múltiplos, o número de visitas domiciliares e o percentual da cobertura de atenção primária estiveram associados à notificação dos casos de TB. Conclui-se que o acesso aos serviços de atenção primária e o aumento das visitas domiciliares são essenciais para ampliar a detecção de casos de TB na cidade do Rio de Janeiro por meio do teste rápido molecular.

## Abstract (en)

Despite efforts to reduce its incidence, tuberculosis (TB) control still challenges the municipality of Rio de Janeiro, Brazil. This study aimed to analyze the temporal trend and effect of TB surveillance and access to health services on the detection of TB cases in Rio de Janeiro (2014-2022). This ecological study evaluated all cases of TB that were reported monthly in residents of Rio de Janeiro. A segmented joinpoint regression model was developed to identify points of change in the trend of notifications and calculate monthly percentage changes. Generalized additive models were used to evaluate the effect of implementing the rapid molecular test, TB surveillance actions, and primary care coverage in the detection of TB cases. The period from January 2014 to December 2022 showed a median of 677 cases of TB per month, with an increasing monthly variation of 0.49% (95%CI: 0.19; 0.79) from January 2014 to August 2017 and of 0.72% (95%CI: 0.16; 1.29) from December 2020 to December 2022. The multiple models associated the number of home visits and percentage of primary care coverage with the notification of TB cases. Access to primary care services and the increase in home visits are essential to expand the detection of TB cases in Rio de Janeiro by the rapid molecular test.

## Abstract (es)

A pesar de los esfuerzos para reducir la incidencia de tuberculosis (TB), su control sigue siendo un desafío para la ciudad de Río de Janeiro, Brasil. Los objetivos de este estudio fueron analizar la tendencia temporal y el efecto de las acciones de vigilancia de la TB y el acceso a los servicios de salud en la detección de casos de TB en la ciudad de Río de Janeiro (2014-2022). Se realizó un estudio ecológico para evaluar todos los casos de TB reportados mensualmente en residentes de la ciudad de Río de Janeiro. Se desarrolló un modelo de regresión *joinpoint* segmentado para identificar puntos de cambio en la tendencia de informes y calcular el cambio porcentual mensual. Se utilizaron modelos aditivos generalizados para evaluar el efecto de la implementación de la prueba molecular rápida, las acciones de vigilancia de la TB y la cobertura de atención primaria en la detección de casos de TB. De enero del 2014 a diciembre del 2022, hubo una mediana de 677 casos de TB por mes, con una variación mensual al alza del 0,49% (IC95%: 0,19; 0,79) de enero del 2014 a agosto del 2017 y del 0,72% (IC95%: 0,16; 1,29) de diciembre del 2020 a diciembre del 2022. En los modelos múltiples, el número de visitas domiciliarias y el porcentual de la cobertura de atención primaria se asociaron con la notificación de casos de TB. El acceso a los servicios de atención primaria y el aumento de las visitas domiciliarias son esenciales para aumentar la detección de casos de TB en la ciudad de Río de Janeiro mediante la prueba molecular rápida.

## Introdução

A tuberculose (TB) se mantém como um importante problema de saúde pública nas Américas, em especial no Brasil, que é o principal país em número absoluto de casos de TB e de coinfecção TB-HIV ^1^. A cidade do Rio de Janeiro, segunda maior cidade do país e polo de atração turística e de negócios, apresentou a terceira maior taxa de incidência de TB do Brasil (95,6 casos/100 mil habitantes) em 2022, atrás apenas de Manaus, Amazonas (115,8 casos/100 mil habitantes) e de Recife, Pernambuco (102,7 casos/100 mil habitantes) ^2^.

Nos últimos anos, houve um esforço global para reduzir a incidência e as mortes por TB no mundo. O Brasil e outros países aderiram a Estratégia End-TB, que estipulou como meta a redução de 20% na taxa de incidência de TB até 2020 e de 50% até 2025, em comparação aos valores de 2015 ^3^. Nesse contexto, houve uma melhora na infraestrutura para o diagnóstico e tratamento de TB no Brasil ^4^. Uma das estratégias para ampliar a detecção dos casos foi a implantação do teste rápido molecular (TRM) Xpert MTB/RIF, com início em 2014, na cidade do Rio de Janeiro e em outros 91 municípios considerados prioritários ^5^. O teste tem elevada acurácia para detecção do *Mycobacterium tuberculosis* ^6^ e, apesar de requerer processamento das amostras em laboratórios com nível de biossegurança NB3, o exame é automatizado e os resultados são liberados em até duas horas ^5^. Posteriormente, devido à baixa carga bacilar observada em indivíduos com coinfecção TB-HIV, em outubro de 2019 essa tecnologia foi substituída pelo Xpert Ultra, capaz de detectar traços do bacilo ^6^. Apesar da melhor sensibilidade, a especificidade pode ficar comprometida pela detecção de bacilos não viáveis em pacientes com histórico de tratamento anterior, o que precisa ser melhor investigado para minimizar potenciais erros na indicação do tratamento. Entretanto, o Xpert Ultra foi plenamente implantado na cidade do Rio de Janeiro a partir de fevereiro de 2020 ^7^.

Em 2020, a pandemia de COVID-19 gerou uma crise sanitária responsável por reverter anos de progresso no combate a TB, particularmente relacionada ao acesso a diagnóstico e tratamento, contribuindo para o atraso no alcance das metas da Estratégia End-TB ^3^. Em 2020, o Brasil teve uma redução de 10,9% no número de casos novos de TB em comparação a 2019, ficando na 12ª posição entre os 16 países com maior redução desse em 2020 ^8^ ^,^ ^9^.

Aliado a essas questões, estudos prévios sugerem que a Estratégia Saúde da Família (ESF) e os programas de transferência de renda impactaram positivamente na incidência e nos desfechos de tratamento de TB no Brasil ^10^ ^,^ ^11^. Entretanto, houve uma mudança nas políticas de financiamento da atenção básica no Brasil, que privilegiou o atendimento por demanda em detrimento da ESF, com reflexos distintos nas diferentes cidades. A reformulação da Política Nacional da Atenção Básica (PNAB) ocasionou uma redução da cobertura da atenção primária no Município do Rio de Janeiro, com a população média coberta indo de 71,1% em 2017 para 46,9% em 2020 ^12^ ^,^ ^13^.

Nesse contexto, o objetivo deste estudo foi analisar a tendência temporal das notificações de TB de 2014 a 2022, bem como avaliar os efeitos da implantação do TRM, da cobertura da atenção primária e das ações de vigilância e controle dessa endemia na detecção de casos de TB na cidade do Rio de Janeiro.

## Métodos

Estudo ecológico para avaliar a tendência temporal das notificações de TB na cidade do Rio de Janeiro, com início em janeiro de 2014, anterior a implantação do TRM, e término em dezembro de 2022.

A redação deste manuscrito seguiu as recomendações da diretriz *Reporting of Studies Using Observational Routinely-collected Data* (RECORD; Relatório de Estudos Usando Dados Observacionais Coletados Rotineiramente), disponível em: http://record-statement.org/checklist.php ^14^.

A cidade do Rio de Janeiro possui uma área de 1.200km^2^, distribuída em 152 bairros. Segundo o Censo Demográfico de 2022, possui 6.211.423 habitantes e uma densidade demográfica de 5.174,7 habitantes/km^2^ ^15^. Em dezembro de 2022, contava com 1.173 equipes de saúde da família e 39 equipes de atenção primária financiadas pelo Ministério da Saúde ^16^.

### Critérios de elegibilidade e fonte de dados

Por fim, foi criada uma variável dicotômica para indicar o início da pandemia de COVID-19: 0 (janeiro de 2014 a janeiro de 2020), 1: (fevereiro de 2020 a dezembro de 2022).

### Análise estatística

Para descrever a série mensal de casos de TB, elaborou-se um gráfico de linha e calculou-se os valores máximo, mínimo e da mediana de notificações. A linha de tendência foi suavizada pelo método Lowess. Essa série foi decomposta para análise de padrões de sazonalidade, tendência e resíduos. A função de autocorrelação e o teste Box-Ljung foram utilizados para avaliar a autocorrelação temporal da série. Para análise da estacionariedade, utilizamos o teste de Dickey-Fuller aumentado ^19^ ^,^ ^20^.

As séries mensais dos procedimentos ambulatoriais, da cobertura de atenção primária e do número de beneficiários de programas de transferência de renda foram descritas por meio de gráficos de linha.

Para identificar os pontos de mudança na tendência de casos de TB, foi construído um modelo de regressão segmentada *joinpoint*, usando a distribuição binomial negativa ^21^ ^,^ ^22^. Foram calculadas a variação percentual anual média (AAPC - *average annual percentage change*) para todo o período, bem como a variação percentual anual (APC - *annual percentage change*) dos três segmentos da linha de tendência com seus respectivos intervalos de 95% de confiança (IC95%) ^23^. Nesse estudo, a unidade temporal foi o mês e, portanto, chamaremos a AAPC e APC de AMPC (*average monthly percentage change*) e MPC (*monthly percentage change*), respectivamente.

Para avaliar o efeito da implantação do TRM, das ações de vigilância de TB, do acesso aos serviços de saúde, dos programas de transferência de renda e da pandemia de COVID-19 (variáveis explicativas) na série mensal de casos de TB (variável desfecho), foram elaborados modelos aditivos generalizados, com a distribuição binomial negativa. Para as variáveis quantitativas não paramétricas, foi adicionado um termo de suavização *spline* na equação de regressão. Inicialmente, foram elaborados modelos simples, para testar o efeito isolado de cada variável explicativa. Nos modelos que avaliaram o efeito da cobertura de atenção primária e do número de beneficiários de programas de transferência de renda, foram adicionadas as variáveis *dummy* indicativas da mudança do método de cálculo ou do programa governamental, respectivamente, e termos de interação. As variáveis que tiveram associação estatística no nível de significância (valor de p < 0,05) foram mantidas no modelo final. Potencial colinearidade entre variáveis foi avaliada pela correlação de Pearson. O ajuste dos modelos foi avaliado por meio do critério de Akaike. Para o modelo final, foi realizada uma análise de resíduos por meio do teste Box-Ljung e função de autocorrelação. As análises foram feitas no programa R (http://www.r-project.org), bibliotecas *stats*, *segmented*, *mgcv* e *ggplot2* ^21^ ^,^ ^24^ ^,^ ^25^ ^,^ ^26^ ^,^ ^27^.

### Considerações éticas

Este estudo utilizou fontes de dados secundários, não identificados, e foi aprovado pelo Comitê de Ética em Pesquisa da Escola Nacional de Saúde Pública Sergio Arouca da Fundação Oswaldo Cruz e da Secretaria Municipal de Saúde (CAAE: 57396922.3.0000.5240).

## Resultados

**Figura 1:**
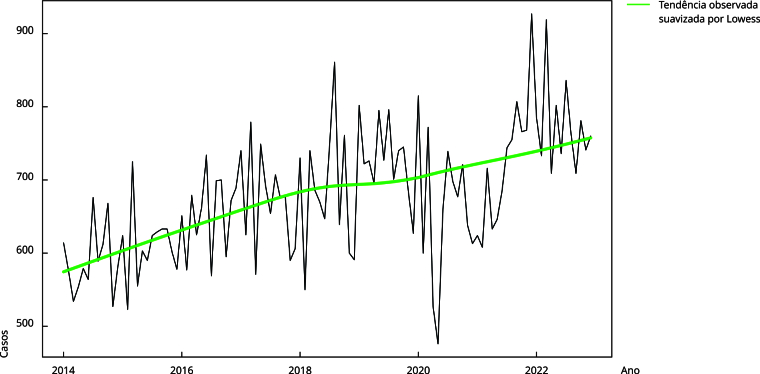
Tendência temporal de casos de tuberculose no Município do Rio de Janeiro, Brasil, janeiro de 2014 a dezembro de 2022.

De janeiro de 2014 a dezembro de 2022, foram notificados 73.182 casos de TB na cidade do Rio de Janeiro, com mediana mensal de 677 casos, mínimo de 476 em maio de 2020, coincidente com a primeira onda de COVID-19, e o valor máximo de 927 casos em dezembro de 2021 (Figura 1). A média trimestral de notificações de TB no segundo trimestre de 2020 (n = 554 casos) foi 33,4% menor em comparação ao segundo trimestre de 2019 (n = 739), com recuperação posterior.

Houve autocorrelação temporal (valor de p < 0,0001 no teste Box-Ljung), com ausência de sazonalidade e de estacionariedade (Dickey-Fuller = 3,2459, *lag order* = 4, valor de p = 0,0839).

**Figura 2:**
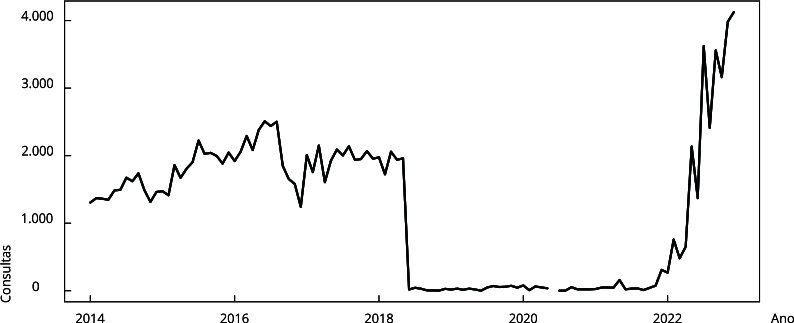
Série temporal do número de consultas com identificação de casos novos de tuberculose no Município do Rio de Janeiro, Brasil, janeiro de 2014 a dezembro de 2022.

**Figura 3:**
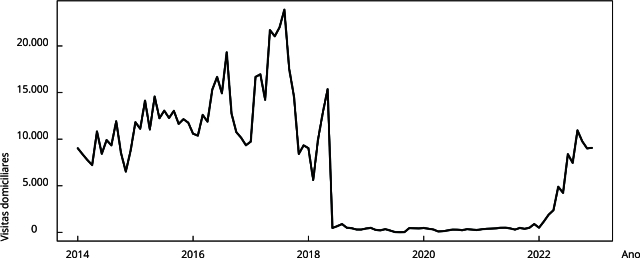
Série temporal do número de visitas domiciliares no Município do Rio de Janeiro, Brasil, janeiro de 2014 a dezembro de 2022.

**Figura 4:**
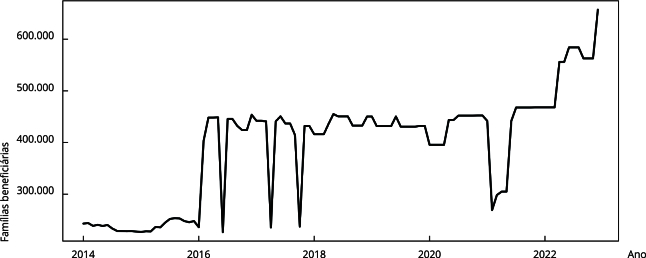
Série temporal do número de famílias beneficiárias de programas de transferência de renda no Município do Rio de Janeiro, Brasil, janeiro de 2014 a dezembro de 2022.

A série de consulta com identificação de casos novos de TB mostrou um aumento de 1.306 procedimentos em janeiro de 2014 para 2.502 em agosto de 2016, com uma redução substancial de 1.960 procedimentos em maio de 2018 para 18 em junho de 2018, permanecendo nesse patamar até setembro de 2021, com recuperação posterior até 4.125 procedimentos em dezembro de 2022 (Figura 2). Observou-se um comportamento semelhante na série mensal das visitas domiciliares (Figura 3). O número de famílias beneficiárias de programas de transferência de renda apresentou uma tendência crescente no período analisado, com uma mediana de 432.155 famílias/mês e máximo de 657.125 em dezembro de 2022 (Figura 4).

**Figura 5:**
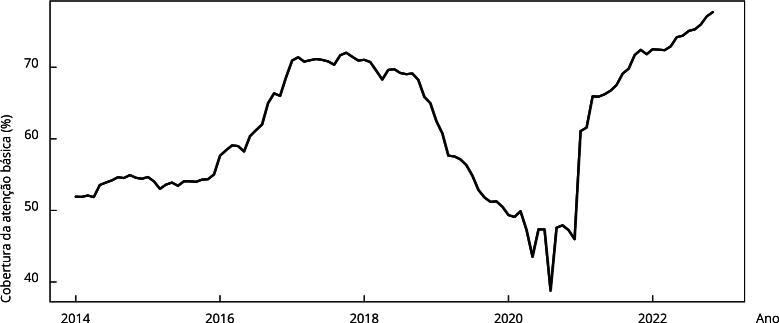
Série temporal do percentual de cobertura de atenção básica no Município do Rio de Janeiro, Brasil, janeiro de 2014 a dezembro de 2022.

A cobertura de atenção primária aumentou de 51,9% em janeiro de 2014 para 70,8% em março de 2017, permanecendo estável até outubro de 2018, com decréscimo subsequente até 38,79% em agosto de 2020 e recuperação posterior até 77,8% em novembro de 2022 (Figura 5).

**Figura 6:**
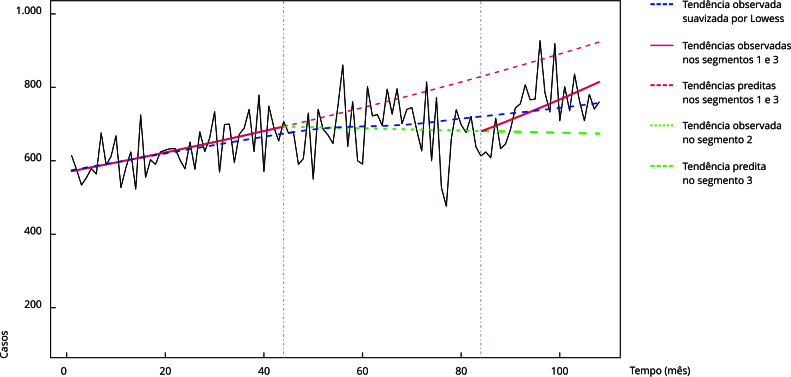
Tendência mensal de notificações de tuberculose no Município do Rio de Janeiro, Brasil, janeiro de 2014 a dezembro de 2022.

A regressão segmentada de *joinpoint* identificou dois pontos de corte na série mensal de notificações de TB: agosto de 2017 e dezembro de 2020 (Figura 6). Ao longo de todo o período, houve uma AMPC nas notificações de 0,0033 (IC95%: 0,0024; 0,0042). No segmento de janeiro de 2014 a agosto de 2017, houve um aumento percentual mensal de 0,4958% (IC95%: 0,1975; 0,7950), com estabilização na detecção de casos até dezembro de 2020 (MPC: 0,0006; IC95%: 0,2292; 0,2285) e posterior aumento a partir desse período (MPC: 0,7241; IC95%: 0,1585; 1,2930).

IC95%: intervalo de 95% de confiança; MPC: variação percentual mensal. Nota: Segmento 1: do mês 0 (janeiro/2014) ao 44 (agosto/2017), MPC: 0,4958, IC95%: 0,1975; 0,7950); Segmento 2: do mês 44 (agosto/2017) ao 84 (dezembro/2020), MPC: -0,0006 (IC95%: -0,2292; 0,2285); Segmento 3: do mês 84 (dezembro/2020) ao final (dezembro/2022), MPC: 0,7241, IC95%: 0,1585; 1,2930).

Nos modelos aditivos generalizados simples, a cobertura de atenção primária (valor de p = 0,0008), a interação com a *dummy* indicadora da mudança do método de cálculo (valor de p *dummy* = 0,0024 e da interação = 0,0039), a pandemia de COVID-19 (valor de p = 0,0002), os procedimentos baciloscopia diagnóstica (valor de p = 0,0011), cultura para BAAR (valor de p = 0,0012) e visita domiciliar por profissional de nível superior (valor de p = 0,0103) mostraram-se estatisticamente significativas. Consulta com identificação de casos novos de TB se mostrou marginalmente significativa (valor de p = 0,0552), mas as respectivas *dummies* e uma das interações foram estatisticamente significativas (valor de p < 0,04). As demais variáveis não apresentaram associação estatisticamente significativa.

**Figura 7:**
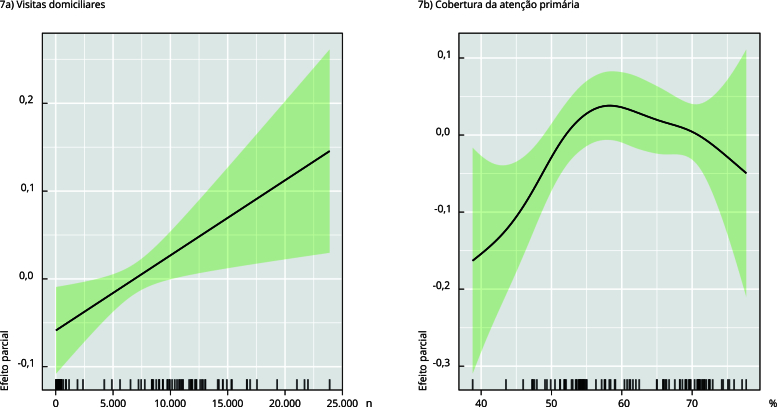
Efeito da cobertura de atenção primária e visitas domiciliares na detecção de casos de tuberculose no Município do Rio de Janeiro, Brasil, janeiro de 2014 a dezembro de 2022.

Quando se adicionou a variável cobertura de atenção primária, apenas os procedimentos de visita domiciliar e consulta com identificação de casos novos de TB permaneceram estatisticamente significativos. Devido à alta correlação entre esses dois procedimentos ambulatoriais (r = 0,81), optamos por incluir apenas visita domiciliar por critério teórico. Os gráficos do modelo final estão mostrados na Figura 7.

Nota: foi adicionado um termo de suavização *spline* às variáveis explicativas s(visita_domicilia) e s(cobertura_ab); equação do modelo aditivo generalizado: mrj~s(mesano) + s(visita_domicilia) + dummy4 + int4 + s(cobertura_ab) + dummy5 + int5 + dummy + int1, data = VARS, family = nb).

Os resultados sugerem que há uma associação temporal direta entre o número de visitas domiciliares e a detecção de casos de TB. Quanto à cobertura de atenção primária, houve uma associação direta com a detecção de casos de TB nos níveis de até 50% de cobertura e uma associação inversa após 70% de cobertura de atenção primária.

O teste Box-Ljung dos resíduos foi marginalmente significativo (valor de p = 0,0450), com uma autocorrelação de primeira ordem muito baixa (r = 0,28).

## Discussão

Os resultados deste estudo mostraram que a cobertura de atenção primária e o número de visitas domiciliares estiveram associados às notificações de TB na cidade do Rio de Janeiro. A pandemia de COVID-19 provocou uma redução temporária na detecção de casos de TB, particularmente na primeira onda epidêmica. Essa redução pode estar relacionada às restrições na circulação de pessoas, orientações de isolamento social, à priorização de atendimentos essenciais e ao direcionamento de recursos financeiros e humanos ao combate da pandemia ^9^ ^,^ ^28^ ^,^ ^29^. Adicionalmente, estudos mostram que o atraso no diagnóstico pode ser devido a certa semelhança entre a sintomatologia respiratória da COVID-19 e TB ^30^ ^,^ ^31^. Porém, essa variação não afetou a tendência de notificações de TB no período do estudo, que se manteve estável de agosto de 2017 a dezembro de 2020, enfatizando o papel da vigilância na identificação dos casos de TB.

A expansão da cobertura de atenção primária na cidade do Rio de Janeiro até 2017 acompanhou a tendência observada no Brasil ^32^. Esses resultados foram consistentes com outros estudos na cidade ^33^ ^,^ ^34^. O aumento na cobertura de atenção primária ocorreu, provavelmente, em consequência aos incentivos fiscais e financeiros federais exclusivamente destinados à expansão e manutenção da ESF, visando fortalecer esse modelo de atenção em saúde. Entretanto, a PNAB regulamentada em 2017 produziu mudanças que propiciaram o financiamento do modelo tradicional de atenção, considerado menos eficiente, utilizando-se como justificativa a crise econômica iniciada em 2014 e o teto de gastos imposto em 2016 ^12^. A PNAB-2017 propôs uma equiparação das equipes do modelo da ESF e do tradicional, que favoreceu a este último ampliar o número de pacientes atendidos por equipe e por agentes comunitários de saúde (ACS), facultar aos gestores municipais a contratação de ACS e flexibilizar a carga horária de médicos e enfermeiros. Como consequência, na cidade do Rio de Janeiro, de 2017 a 2020 houve uma redução relativa na média anual de cerca de 22% no número de ACS, de 85% no quantitativo de visitas domiciliares por mil habitantes, de 81% de consultas médicas e de 82% nas consultas de enfermagem, ambas de adultos e por mil habitantes, em comparação ao período de 2010 a 2017 ^12^.

Em nosso estudo, a redução na cobertura de atenção primária observada a partir março de 2017 coincidiu com a implantação da PNAB-2017. Essa tendência ficou mais evidente a partir de 2018, acompanhada de uma queda brusca no número de visitas domiciliares, coincidindo com a reorganização dos serviços de atenção primária em saúde na cidade do Rio de Janeiro alinhada à PNAB-2017 ^35^.

Os resultados mostraram uma associação direta entre a cobertura de atenção primária e o número de visitas domiciliares com as notificações de TB na cidade do Rio de Janeiro, até aproximadamente 50%, com uma associação inversa a partir de 70% de cobertura de atenção primária, porém sem significância estatística, provavelmente devido ao número reduzido de meses com níveis elevados de cobertura APS na cidade do Rio de Janeiro. Além disso, mostraram uma associação direta e linear com o número mensal de visitas domiciliares. A expansão da atenção primária na cidade do Rio de Janeiro e o crescente aumento no número de visitas domiciliares provavelmente influenciaram positivamente a detecção de casos de TB observada de janeiro de 2014 a agosto de 2017. Porém, a mudança na política de atenção básica em 2017 pode ter impactado negativamente a busca ativa por meio das visitas domiciliares, com estabilização na detecção de casos de TB. Esses resultados reforçam a importância das políticas públicas eficientes para o controle da TB, de preferência em conjunto com outros setores de saúde (p.ex.: HIV/aids, saúde mental) para ampliar o acesso ao diagnóstico precoce e a implementação oportuna do tratamento, bloqueando a transmissão dessa doença.

A implantação do TRM em dezembro de 2014 ^5^, com mudança do Xpert para o Ultra em fevereiro de 2020 ^7^, não foi suficiente para aumentar a detecção de casos de TB nos níveis esperados, comparada à cobertura de atenção primária e às visitas domiciliares. Em pouco tempo de implantação, potenciais problemas relacionados ao abastecimento regular e equitativo deste insumo diagnóstico para as unidades e à disponibilidade do teste para casos suspeitos e seus contatos domiciliares puderam ser consideradas. Por outro lado, a entrada da tecnologia diagnóstica, ainda que de alta acurácia, requer políticas públicas que garantam estratégias efetivas de captação de indivíduos suspeitos para que o diagnóstico e a notificação dos casos sejam possíveis.

Este estudo apresenta forças e limitações. Ao que sabemos, não há estudos que avaliaram o efeito das ações de saúde e de políticas sociais, aliadas à introdução das novas tecnologias diagnósticas, na detecção de casos de TB. Portanto, este é o primeiro que se propõe avaliar a efetividade dos testes diagnósticos moleculares, considerando o impacto da mudança das políticas públicas na cobertura de atenção primária e na busca ativa de casos de TB. Adicionalmente, este é um estudo de base populacional, pois considerou todos os casos notificados de TB em residentes da cidade do Rio de Janeiro. O atendimento a essa doença é feito prioritariamente nas unidades públicas de saúde, que têm exclusividade na dispensação dos medicamentos específicos para o tratamento da TB. Por outro lado, o atraso e a subnotificação de casos são sempre possíveis ^36^ ^,^ ^37^. Porém, isso deve ter sido minimizado ao considerar um longo período de avaliação, pelas sucessivas extrações para atualização dos dados e das análises, aliado ao fato da dispensação do tratamento ser feita exclusivamente nas unidades de atenção primária e vinculada à notificação. Uma limitação deste estudo está relacionada ao uso do indicador cobertura de atenção primária, que representa uma das dimensões do acesso aos serviços de saúde. Um estudo sugere que a descentralização das ações de TB para o Programa Saúde da Família não foi capaz de reduzir as desigualdades de acesso ao diagnóstico para a população masculina, de baixa renda, em situação de rua ou privada de liberdade, que são as de maior vulnerabilidade para essa doença ^38^. Outra limitação se refere ao desenho ecológico, que dificulta afirmar sobre uma associação causal. Entretanto, possibilita analisar o efeito contextual das políticas e das ações de saúde e, dessa forma, identificar falhas no processo que devam ser enfrentadas para ampliar o acesso ao diagnóstico da TB em áreas endêmicas. Apesar do estudo estar limitado à capital do Rio de Janeiro, é plausível supor que estes achados sejam aplicáveis a outras metrópoles brasileiras com características similares em termos de situação socioeconômica, de vigilância e organização de serviços para o controle da TB.

Como conclusão, o número de visitas domiciliares e a cobertura de atenção básica ou primária foram os fatores determinantes para a detecção de casos de TB na cidade do Rio de Janeiro. Estes resultados reforçam a importância das estratégias de ampliação do acesso aos serviços de saúde, e do aumento da suspeição clínica e busca ativa de casos, em especial dos subgrupos mais vulneráveis da população, para o alcance das metas de detecção de TB. As novas tecnologias diagnósticas, como o Xpert MTB/Rif, favorecem a detecção oportuna, entretanto, sem haver um paralelismo com as melhorias do nível socioeconômico e de infraestrutura de serviços de saúde para alcançar as populações mais vulneráveis à tuberculose, as falhas no controle da TB se perpetuarão.

## Data Availability

Foram incluídas todas as notificações de casos novos de TB de residentes do Município do Rio de Janeiro, registrados na base municipal do Sistema Nacional de Agravos de Notificação (SINAN; http://tabnet.rio.rj.gov.br/cgi-bin/dh?sinan/definicoes/tuberc2007.def), para o período do estudo. Foi criada uma variável qualitativa para determinar a implantação dos dois TRM, com as seguintes categorias: 0 (ausência de TRM, janeiro a novembro de 2014), 1 (Xpert MTB/Rif, dezembro de 2014 a janeiro de 2020), e 2 (Xpert Ultra, fevereiro de 2020 a dezembro de 2022). As ações de vigilância voltadas para a detecção de casos de TB foram avaliadas pelos seguintes procedimentos ambulatoriais, informados pelas unidades municipais, cujos dados foram extraídos da base nacional do Sistema de Informações Ambulatoriais do Sistema Único de Saúde (SIA/SUS; http://tabnet.datasus.gov.br/cgi/deftohtm.exe?sia/cnv/qarj.def): 0101030029 - Visita domiciliar/institucional por profissional de nível superior (visita); 0301010021 - Consulta com identificação de casos novos de tuberculose (consulta_id); 0202080048 - Baciloscopia direta para bacilo álcool-ácido resistente (BAAR) (diagnóstica); 0202080110 - Cultura para BAAR e 0204030170 - Radiografia de Tórax (PA). A cobertura de serviços de saúde foi avaliada por meio do percentual da população coberta pela atenção básica na cidade do Rio de Janeiro, extraído dos Painéis de Indicadores da Atenção Primária, da Secretaria de Atenção Primária à Saúde do Ministério da Saúde. Os dados para cobertura de atenção básica (janeiro de 2014 a dezembro de 2020) estão disponíveis em: https://sisaps.saude.gov.br/painelsaps/saude-familia, conforme definido pela *Portaria nº 703/2011* ^17^. De janeiro de 2021 a dezembro de 2022, o indicador utilizado foi a cobertura de atenção primária, disponível em https://sisaps.saude.gov.br/painelsaps/cobertura_aps, em conformidade com as diretrizes da PNAB-2019, *Portaria nº 2.539/2019* ^18^. Para considerar a mudança de cálculo desse indicador a partir de janeiro de 2021, foi gerada uma variável *dummy* (0: janeiro de 2014 a dezembro de 2020; 1: janeiro de 2021 a dezembro de 2022).
